# Gene-expression profiles of abdominal perivascular adipose tissue distinguish aortic occlusive from stenotic atherosclerotic lesions and denote different pathogenetic pathways

**DOI:** 10.1038/s41598-020-63361-5

**Published:** 2020-04-10

**Authors:** Luca Piacentini, Claudio Saccu, Elisa Bono, Elena Tremoli, Rita Spirito, Gualtiero Ivanoe Colombo, José Pablo Werba

**Affiliations:** 1 Immunology and Functional Genomics Unit, Centro Cardiologico Monzino, IRCCS, 20138 Milan, Italy; 2 Atherosclerosis Prevention Unit, Centro Cardiologico Monzino, IRCCS, 20138 Milan, Italy; 3 Vascular and Endovascular Surgery Unit, Centro Cardiologico Monzino, IRCCS, 20138 Milan, Italy; 4 Scientific Direction, Centro Cardiologico Monzino, IRCCS, 20138 Milan, Italy

**Keywords:** Aortic diseases, Atherosclerosis

## Abstract

Perivascular adipose tissue (PVAT) helps regulate arterial homeostasis and plays a role in the pathogenesis of large vessel diseases. In this study, we investigated whether the PVAT of aortic occlusive lesions shows specific gene-expression patterns related to pathophysiology. By a genome-wide approach, we investigated the PVAT transcriptome in patients with aortoiliac occlusive disease. We compared the adipose layer surrounding the distal aorta (atherosclerotic lesion) with the proximal aorta (plaque-free segment), both *within* and *between* patients with complete aortoiliac occlusion (Oc) and low-grade aortic stenosis (St). We found that PVAT of the distal versus proximal aorta *within* both Oc- and St-patients lacks specific, locally restricted gene-expression patterns. Conversely, singular gene-expression profiles distinguished the PVAT *between* Oc- and St-patients. Functional enrichment analysis revealed that these signatures were associated with pathways related to metabolism of cholesterol, vessel tone regulation, and remodeling, including TGF-β and SMAD signaling. We finally observed that gene-expression profiles in omental-visceral or subcutaneous fat differentiated *between* Oc- and St-patients, suggesting that the overall adipose component associates with a different atherosclerosis burden. Our work points out the role of PVAT and, likely, other adipose tissues play in the pathophysiological mechanisms underlying atherosclerotic disease, including the abdominal aortic occlusive forms.

## Introduction

Common pathologies of the distal abdominal aorta include the dilated (aneurysmal) and the obstructive (atherosclerotic) forms^1,2^. Although the two conditions share some vascular risk factors (*eg*, cigarette smoking, hypertension, dyslipidemia), both epidemiology^3,4^ and pathogenesis^5–7^ of the diseases are clearly different. Even so, increasing evidences suggest that dysfunctional perivascular adipose tissue (PVAT) participates in the development and progression of both atherosclerotic and nonatherosclerotic vascular diseases^8,9^, including those affecting the distal abdominal aorta.

PVAT is known to influence artery homeostasis by tuning many physiological functions, which comprise the regulation of vessel tone through the activation of pro- or anti-contractile mechanisms, secretion of soluble factors, and modulation of local inflammation^10^, and its dysfunction may adversely affect vessel health. PVAT is highly heterogeneous and its pathophysiological roles may be different depending on the segments of the artery beds^11^.

Animal models of peripheral artery disease often provide findings that translate poorly to patients^12^, prompting human specimens to be used as crucial for vascular research. The availability of tissue samples from patients with vascular diseases is declining rapidly due to the increasing shift from vascular surgery to endovascular repair^13–15^, making the samples collected after surgery particularly valuable. They represent, hence, a unique source of information for molecular profiling studies. In particular, genome-wide expression profiling allows exploring the molecular landscape of diseased tissue without the need for an a priori selection of factors to be studied and, therefore, potentially able to reveal new mechanisms of disease onset or progression^16^.

Recently, we have shown that disease-specific gene expression patterns are locally-restricted to the PVAT surrounding abdominal aortic aneurysms (AAA) in human patients, that these molecular signatures are suggestive of an autoimmune response, and that changes in gene expression have increased in number and magnitude with disease extent and progression^17^.

To date, large-scale gene expression studies examining the role of PVAT in the atherosclerotic disease of the abdominal aorta are lacking.

In the present study, we tested through a genome-wide approach whether the PVAT localized at the site of atherosclerotic lesions of the distal aorta showed disease-specific gene expression profiles and whether there were expression patterns associated with the disease severity. To this end, we enrolled peripheral artery disease (PAD) patients, presenting with an aortoiliac occlusive disease (AIOD) or diffuse stenosis of the aorta and common iliac artery (CIA). We performed analyses by a (1) ‘*within* patients’ comparison of the transcriptome of PVAT surrounding the atherosclerotic-affected distal aorta with that of the abdominal aorta’s lesion-free proximal segment, and an analysis (2) ‘*between* patients’ of PVAT gene expression profiles of a complete aortoiliac occlusion versus subjects with low-grade aortic stenosis. We used an up-to-date data mining procedure, which increases the overall sensitivity and robustness of the analysis by controlling for unwanted sources of variation^18,19^. Based on the resulting gene expression signatures, we inferred underlying pathogenic mechanisms.

## Results

### Patient characterization

The Table 1 reports patients’ characteristics. PAD patients can present a different degree of obstruction at the site of the abdominal aorta. In compliance with the Trans-Atlantic Inter-Society Consensus Document on Management of Peripheral Arterial Disease (TASC) classification^20^, we divided patients having type D lesion into two groups: (i) infra-renal aortoiliac occlusions (Oc, n = 6) and (ii) unilateral or bilateral CIA occlusions or diffuse multiple stenoses involving CIA (with or without stenosis of the distal aorta; St, n = 5). The distinction was made by detailed visual analysis of a contrasted tomography carried out within two months before the surgical procedure. The above-mentioned patients’ sub-classification mirrored their anamnestic personal history defined by the timing of referred symptoms (intermittent claudication of thighs and legs) that was significantly lower in Oc compared to St-patients (*P* value = 0.02; see Table 1). There were, instead, no other significant differences in clinical parameters and medications between Oc- and St-patients (*ie*, *P* value > 0.05).

**Table 1:** Patient clinical characteristics.

Demographics	Total patients (n = 11)	Occlusive (n = 6)	Stenotic (n = 5)
Age (years)	62 (58–69)	59 (57–61)	66 (64–72)
Sex (M/F)	9/2	5/1	4/1
Timing to referred symptom (years)*	6 (2.5–12.0)	2.5 (1.2–3.7)	14 (7.0–23.0)
BMI (kg/m^2^)	25.5 (24.0–28.2)	25.2 (24.8–27.8)	25.7 (23.0–27.7)
CHD	6 (55%)	3 (50%)	3 (60%)
CeVD	2 (18%)	2 (33%)	0
COPD	2 (18%)	1 (17%)	1 (20%)
Hyperuricemia	1 (9%)	1 (17%)	0
Type 2 Diabetes	3 (27%)	2 (33%)	1 (20%)
CKD	2 (18%)	0	2 (40%)
**Risk factors**			
Smokers	5 (45%)	2 (33%)	3 (60%)
Past smokers	6 (55%)	4 (67%)	2 (40%)
Hypertension	7 (64%)	3 (50%)	4 (80%)
SBP	128 (120–146)	126 (120–130)	134 (110–150)
DBP	80 (69–80)	80 (80–80)	70 (60–80)
Dyslipidemia	8 (73%)	4 (67%)	4 (80%)
**Laboratory tests**			
Total cholesterol (mg/dL)	186 (164–224)	178 (163–205)	200 (186–235)
HDL-C (mg/dL)	41 (40–80)	47 (40–93)	40 (40–46)
Triglycerides (mg/dL)	137 (116–166)	132 (126–139)	150 (88–182)
**Medications**			
Antiaggregants	10 (91%)	6 (100%)	4 (80%)
Diuretics	4 (36%)	3 (50%)	1 (20%)
Beta blockers	3 (27%)	2 (33%)	1 (20%)
Calcium blockers	3 (27%)	1 (17%)	2 (40%)
ACE inhibitors	2 (18%)	1 (17%)	1 (20%)
ARBs	4 (36%)	2 (33%)	2 (40%)
Oral hypoglycemics	1 (9%)	1 (17%)	0
Statins	5 (45%)	2 (33%)	3 (60%)
Proton pumps inhibitors	7 (64%)	4 (67%)	3 (60%)
No drugs	1 (9%)	0	1 (20%)

Categorical variables are shown as counts (*n*) and percentage (%); quantitative variables are expressed as the median and interquartile range (Q1–Q3). BMI, body mass index; CHD, coronary heart disease; CeVD, cerebrovascular disease; COPD, chronic obstructive pulmonary disease; CKD, chronic kidney disease; SBP, systolic blood pressure; DBP, diastolic blood pressure; HDL-C, high-density lipoprotein cholesterol; ACE, angiotensin-converting-enzyme; ARBs, angiotensin II receptor blockers. *Timing to referred symptom variable displayed a significant difference between Oc vs St (P-value = 0.02). None of the other variables showed significant differences (*ie, P*-value > 0.05) between Oc- and St-patients: comparisons were made by a two-sided Wilcoxon test or Fisher’s exact test for continuous and categorical variables, respectively.

### Gene expression dataset

Following the probe-filtering criteria, we identified 18149 expressed probes. After re-annotation, we obtained a final expression matrix of 14352 transcripts, which correspond to 11105 unique genes (annotation details in the online-only Data Supplement 2).

### Adipose tissue samples clustering by principal component analysis (PCA) on whole gene-expression data

**Figure 1:**
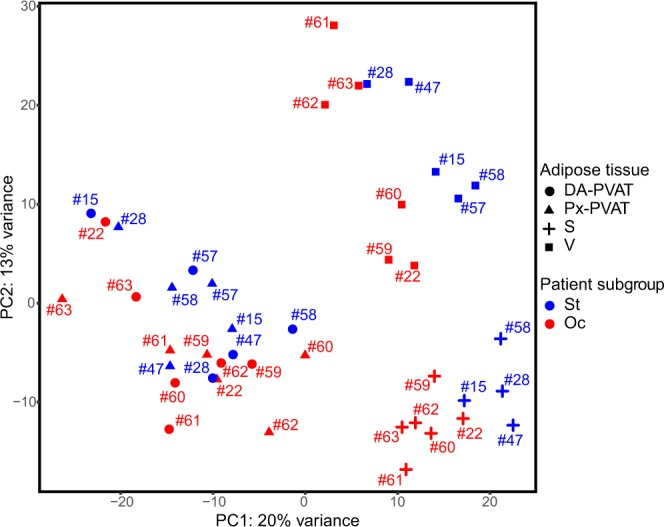
Unsupervised clustering of AT samples by PCA. Scatterplot of the first two principal components (PC1 and PC2) obtained from the PCA performed on the whole gene-expression dataset. PC1 and PC2 explained together 33% variance and allowed discriminating the different AT samples. Patients with aortic occlusive (red) and stenotic lesions (blue) tend to form two distinct sub-clusters within each AT. Circle, triangle, plus and square shapes associate, respectively, with DA-PVAT, Px-PVAT, subcutaneous (S) and omental-visceral (V) AT. Numbers refer to patient’s paired-samples.

We used the entire, adjusted gene-expression matrix of PVAT, subcutaneous and omental-visceral adipose tissue (AT) samples of the PAD patients to get an overview of the expression data and inspect possible sub-sample grouping. As expected, the scatterplot of the two first principal components of the PCA, which together explain the 33% of the variance, showed that samples unambiguously clustered based on the type of AT depot. Furthermore, we observed a trend towards a distinction between samples of patients with complete occlusive lesions compared to those with stenotic lesions within PVAT and, intriguingly, a clearer separation within subcutaneous and omental-visceral AT samples (Fig. 1).

### Differential expression analysis of PVAT of the distal versus the proximal aorta within patients with occlusive or stenotic lesions

**Figure 2:**
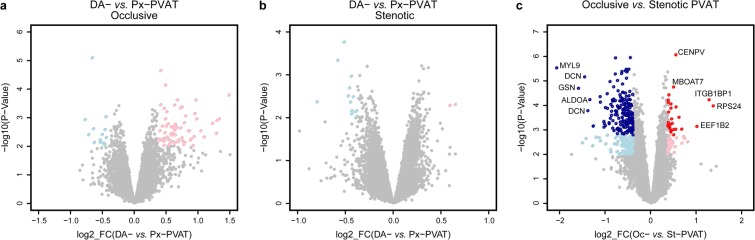
PVAT differential expression analysis. Scatterplot of the log2 FC vs. the significance (x-and y-axis, respectively) for the paired DA-PVAT vs. Px-PVAT comparison within patients with aortic occlusive (**A**) or stenotic (**B**) lesions, and for the comparison between PVAT of patients with aortic occlusive vs. stenotic lesions (**C**). Significant DE transcripts with an absolute log2 FC ≥ 0.38 at nominal P-Value < 0.01 are represented by pink and light blue dots, whereas red and dark blue dots mark the transcripts that stood adjustment for multiple testing (adj.P-Value < 0.05). For the latter comparison, the top five up/down DE transcripts with the highest combined rank-score (the product of the log2 FC and the -log10 P-value) are shown.
(III). *DASt - versus PxSt-PVAT*: we observed 15 DE transcripts (nominal *P* value < 0.01) with |log_2_FC | ≥0.38, ranging from 0.64 to –0.80. Two transcripts were over- and 13 under-expressed in DASt- compared to PxSt-PVAT, respectively (Fig. 2B).

First, we performed a paired-samples analysis of distal (DA) versus proximal (Px) abdominal aorta *within* all patients, *i*e DA- *vs*. Px-PVAT. Second, since our patients present a different grade of atherosclerotic lesions, we also performed differential expression analysis after splitting subjects with total occlusive lesions from those with stenotic lesions, *i.e*. DAOc- *vs*. PxOc-PVAT and DASt- *vs*. PxSt-PVAT. We reported the overall results for all the three comparisons in the online-only Data Supplement 2A. Briefly, the main findings are:

(I). *DA- versus Px-PVAT on total samples:* we detected 10 differentially expressed (DE) transcripts (nominal *P* value < 0.01) with absolute log_2_ fold-change (|log_2_FC | ) ≥0.38, ranging from 0.75 to –0.44. (Figure I in the online-only Data Supplement 1). Seven transcripts were over- and 3 under-expressed in DA- compared to Px-PVAT, respectively.
(II). *DAOc- versus PxOc-PVAT*: we found 79 DE transcripts (nominal *P* value < 0.01) with |log_2_FC | ≥0.38, ranging from 1.48 to –0.66. Among them, 66 and 13 transcripts were over- and under-expressed in DAOc- compared to PxOc-PVAT, respectively (Fig. 2A).

Nonetheless, none of these DE transcripts stood correction for multiple testing, in any comparison. Consistently, the histograms of the *P*-value distributions did not fit that expected for truly DE genes (Figure IIA,B,C in the online-only Data Supplement 1). These results indicate a lack of substantial locally-restricted differences in the PVAT of the distal aorta compared to the proximal abdominal aorta, both for patients with occlusive and stenotic lesions.

### Comparison of PVAT of the abdominal aorta between patients with occlusive versus stenotic abdominal aortic lesions

AT samples clustering by PCA on the global gene expression profiles (Fig. 1) suggested that the abdominal aortic PVAT could present relevant differences *between* the two subgroups of PAD patients. Indeed, differential expression analysis comparing PVAT in Oc- *vs*. St-patients revealed 210 DE transcripts with |log_2_FC | ≥0.38, ranging from 1.38 to –2.06, that stood correction for multiple testing at an false discovery rate (FDR)-adjusted *P*-value < 0.05. Among them, 26 and 184 transcripts were over- and under-expressed in Oc- compared to St-PVAT, respectively (Fig. 2C and online-only Data Supplement 2b). Consistently, the histogram of the *P* values distribution displayed the expected shape for truly DE transcripts (Figure IID in the online-only Data Supplement 1).

**Figure 3:**
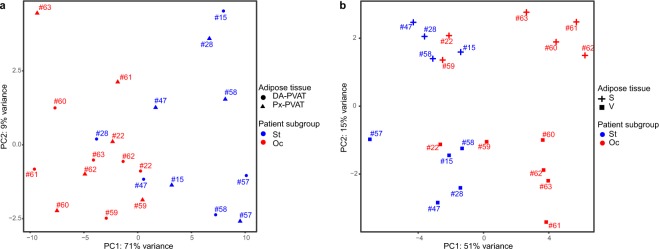
Unsupervised clustering of AT samples by PCA based on PVAT DE transcripts. Scatterplot of the first two principal components (PC1 and PC2) obtained from the PCA performed on the expression matrix of the 210 DE transcripts obtained by comparing Oc *vs*. St-PVAT, for PVAT samples (**A**) and omental-visceral (V), subcutaneous (S) AT (**B**). PC1 and PC2 explained together 80% and 66% variance for PVAT and V-S depots, respectively, allowing discriminating most of the Oc- vs. St-patients. Red and blue colors represent Oc- and St-patients, respectively. Circle, triangle, plus and square shapes associate, respectively, with DA-PVAT, Px-PVAT, subcutaneous and omental-visceral AT. Numbers refer to patient’s paired-samples.

We then plotted the first two principal components of a PCA based on the expression values of the above mentioned 210 DE transcripts and observed an almost complete distinction between Oc- and St-PVAT samples, from both the distal (diseased) and proximal (plaque-free) segments of the abdominal aorta (Fig. 3A). Interestingly, unsupervised clustering by applying a PCA to omental-visceral and subcutaneous AT based on these 210 DE transcripts also provided good separation between samples of patients with occlusive lesions and those with stenotic lesions (Fig. 3B).

### Comparison of omental-visceral and subcutaneous AT between patients with occlusive versus stenotic abdominal aortic lesions

PCA results in Figs. 1 and 3B prompted us to examine whether group-specific differences also exist between the omental-visceral and subcutaneous AT depots of Oc- *versus* St-patients (online-only Data Supplement 2B). An *ad hoc* differential expression analysis of Oc *vs*. St revealed 89 DE transcripts in omental-visceral AT (at a nominal P value < 0.01 and |log_2_FC | ≥0.38, ranging from 1.59 to –1.35), with 14 and 75 overexpressed transcripts in Oc- and St-subgroup, respectively (Figure IIIA in the online-only Data Supplement 1). For subcutaneous AT, we observed 190 DE transcripts (at a nominal P value < 0.01 and |log_2_FC | ≥0.38, ranging from 1.59 to –1.35) with 32 and 158 overexpressed transcripts in Oc- and St-subgroup, respectively (Figure IIIB in the online-only Data Supplement 1). In the latter analysis, 3 DE transcripts stood correction for multiple testing (FDR-adjusted *P* value < 0.05). However, for both comparisons, the histograms of the *P*-value distribution showed a clear trend towards the expected shape for truly DE transcripts (Figure IIIC,D in the online-only Data Supplement 1).

### Functional inferences from genome-wide differential expression analysis of Oc- versus St-PVAT

**Figure 4:**
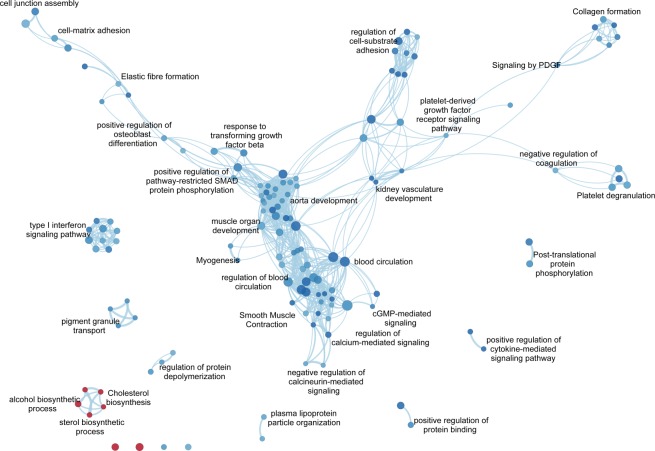
Enrichment network for Oc- vs.St-PVAT comparison. The enrichment network shows the pathway/GO-BP gene-sets (nodes) that are significantly associated (FDR < 0.05) either with Oc- *or* St-PVAT. The node color refers to the association with the phenotype (Oc-PVAT, red; St-PVAT, blue); node gradient color is proportional to the gene-set normalized enrichment score (NES), from lower (light) to higher (dark); node size is proportional to the gene-set size. Edges connect related pathways/GO-BPs. Edge thickness is proportional to the similarity between two pathway/GO-BP, for a cut-off = 0.15 of the combined Jaccard plus Overlap coefficient. To simplify network reading, only relevant gene-sets are labeled with the name reported in Reactome or GO-BP gene-set collection. Enrichment network was drawn using the Enrichment Map software v.3.2.0, implemented as a plug-in in the Cytoscape v.3.7.0 platform.

We tested which Gene Ontology (GO) Biological Processes (BP)/pathways were associated with the Oc- and St-PVAT phenotypes by Gene Set Enrichment Analysis (GSEA). We found that 7 and 129 GO-BP/pathways were positively associated with the Oc-PVAT and the St-PVAT samples, respectively (FDR *q* value < 0.05; see online-only Data Supplement 3A). We further summarized GSEA results into an enrichment network to visualize the relationships among the most relevant GO-BP/pathway gene-sets and help data interpretation (Fig. 4). GO-BP/pathways associated with Oc-PVAT mainly related to cholesterol, sterol, and alcohol biosynthetic processes. Conversely, the most interconnected GO-BP/pathways upregulated in St-PVAT involved those related to muscle and circulatory system processes (including smooth muscle contraction and aorta development), regulation of blood circulation, regulation of cell-substrate adhesion and cell junction assembly, collagen and elastic fiber formation, cell-matrix adhesion, negative regulation of coagulation and platelet degranulation. Among signaling pathways, we observed significant associations of St-PVAT with those linked to platelet-derived growth factor (PDGF), cyclic guanosine monophosphate (cGMP), type I interferon, response to TGF (transforming growth factor) beta, and positive regulation of pathway-restricted SMAD protein phosphorylation, as well as with positive regulation of cytokine-mediated signaling pathway and regulation of calcium and calcineurin-mediated signaling.

### Functional inferences from genome-wide differential expression analysis of Oc- versus St in omental-visceral and subcutaneous AT depots

We finally performed GSEA for specific associations of GO-BP/pathways with Oc *versus* St (*i*) omental-visceral and (*ii*) subcutaneous AT (online-only Data Supplement 3B and 3C). We summarized GSEA results as enrichment networks in Figures IV and V in the online-only Data Supplement 1. The main findings are:

(I). *Oc vs. St-Omental-visceral AT*: we observed 123 gene-sets significantly associated with the St samples that are largely related to fatty acid metabolism, cellular respiration, transcriptional regulation of white adipocyte differentiation, and immune-related functions involving both the innate and adaptive immune response.
(II). *Oc vs. St-Subcutaneous AT:* twenty-five and 44 gene-sets were associated with Oc and St samples, respectively. Significant GO-BP/pathways characterizing Oc samples included eukaryotic translation, granulocyte migration, and response to chemokine, whereas those related to St samples encompassed fatty acid metabolism, low-density lipoprotein receptor particle metabolic process, Golgi associated vesicle biogenesis, and regulation of ERBB/EGFR (epidermal growth factor receptor) signaling pathway.

## Discussion

PVAT is a well-recognized regulator of vessel homeostasis and its dysfunction may strongly influence the pathogenesis of vascular diseases^21,22^. Herein, we tested on a genome-wide scale whether distinctive gene expression patterns were associated with the PVAT surrounding occlusive and stenotic segments of the abdominal aorta in PAD patients. Our study showed that perilesional aortic PVAT in PAD patients lacks specific gene expression signatures, as we did not observe any significant DE gene compared to the PVAT of the plaque-free segments in subjects with either occlusive or stenotic disease. This indicates that in PAD the entire PVAT of the abdominal aorta had uniform gene expression profiles with no evident locally restricted differences in the diseased segment. On the contrary, the PVAT of the abdominal aorta displayed gene expression signatures distinguishing PAD patients with a different burden of atherosclerotic lesions, *ie*, patients with occlusive *versus* sub-occlusive lesions. Furthermore, unsupervised clustering analysis of AT samples by PCA showed a clear, not obvious separation of PAD patients with occlusive from those with stenotic lesions for all the AT depots considered. This suggests that subgroups of patients with PAD that differ in severity of abdominal aortic disease may show significant different expression profiles at the systemic level involving other AT depots.

Consistent with this hypothesis, we identified three main biological processes that characterize the AT of these two forms of PAD, with possible pathogenetic implication: (i) metabolism of lipids (*ie* cholesterol and fatty acids), (ii) maintenance/remodeling of the arterial vessel and (iii) involvement of immune response.

The lipid component may play a role in the pathogenesis of the two forms of PAD through different mechanisms. Accumulation and modification (aggregation, oxidation or enzymatic cleavage) of cholesterol in the sub-endothelial layer of the arteries is a well-known mechanism to produce pro-atherogenic cholesterol particles. Over-representation of cholesterol biosynthetic pathways in the PVAT of Oc-patients suggests that changes in its metabolism^23^ may result in a strong pro-atherogenic effect that may produce a severe and faster occlusion of the aorta in these patients. Conversely, the association of St-patients with the “fatty acid metabolism” pathway in both the omental-visceral and subcutaneous AT and the “low-density lipoprotein particle metabolic process” in the subcutaneous AT is intriguing, since both the visceral and subcutaneous AT are energy storage tissues and lipid catabolic regulation can be triggered to reduce the effects of atherogenic stimuli, as demonstrated in *in vivo* animal models^24^. Moreover, PAD has been suggested to have a unique lipoprotein signature compared to coronary and cerebrovascular diseases, mainly characterized by increased low-density lipoprotein particles rather than LDL cholesterol content^25^. As AT are also known to regulate serum lipids^26^, reducing LDL particles through upregulation of the LDL particle receptor in AT may represent a feedback mechanism to lessen circulating LDL particles in St-patients, similar to what happens with the use of lipid-lowering therapies^27^. An exhaustive assessment of the lipid profile, including standard lipid concentrations, lipoproteins (*eg*, LDL particles), and proteins affecting lipoprotein homeostasis (*eg*, PCSK9), could provide a deeper insight into the role of atherogenic lipids and lipoproteins in these two types of PAD.

The second element which may account for a different pathogenesis between Oc- and St-patients concerns the maintenance and remodeling of the vessel structure and the control of the vessel tone. We may hypothesize that a long-lasting atherosclerotic process, which characterize our St-patients, may trigger an adaptive response that tries to maintain the balance between arterial function and structure. Indeed, St-patients over-expressed genes related to pathways of elastic fiber and collagen formation, aorta and muscle structure development and contraction, and regulation of cell-substrate adhesion that may play a relevant role to counteract a vessel injury^28^. This is consistent with the knowledge that PVAT has either pro- or anti-atherosclerotic properties^29^. In this context, “TGF (transforming growth factor)-beta response”, which was interconnected with the above-mentioned pathways, is known to have athero-protective effects and tissue repair properties^30^. Moreover, TGF-beta also exert its pro-fibrotic effects through SMAD signaling to induce matrix-related genes, such as collagens, fibronectin, plasminogen activator inhibitor, and proteoglycans (see functional network; Fig. 4). Interestingly, recent findings showed that PVAT-derived mesenchymal stem cells contribute to vascular remodeling *in vivo* through smooth muscle cell differentiation and metabolic reprogramming promoted by TGF-signaling and specific microRNA regulation^31^. Further investigations for histological analysis of the full-thickness aorta to find marker of fibrosis and extracellular matrix remodeling may strengthen the hypothesis that TGF-beta signaling may have indeed a distinct pathogenic role in Oc- versus St-patients.

Finally, the omental-visceral AT of St-patients showed over-represented pathways related to immune functions, including both innate and adaptive response. Both the omentum and subcutaneous adipose tissue are populated with immune cells and are associated with adverse metabolic risk factors, although the omental-visceral AT is recognized to play a major role^32–34^. Since the activity of immune cells in AT can affect adjacent tissues and organs^35^, we may hypothesize that the omental AT of St-patients triggers an adaptive response that attempts to counteract an evolving atherosclerotic process. Testing if Oc-and St-patients have a different immune profile, *eg* by examining their T- and B-cell receptor repertoires in these ATs and/or systemic blood circulation, would provide new insights into the role of various components of the immune system.

To our knowledge, this is the first study that describes the transcriptome of PVAT in PAD patients with AIOD or diffuse stenosis of the aorta and CIA through a genome-wide approach and has several strengths.

First, transcriptome analysis is an effective approach to reveal even subtle changes due to environmental stimuli, genetic and epigenetic background, and a different pathophysiological status. Furthermore, it favors applying a data-driven strategy that often allows identifying unexpected findings and new interpretations^16^. Second, the use of a paired-data approach reduces the effects of heterogeneity among subjects and increases the overall sensitivity and statistical power of the analysis^18^. Third, the state-of-the-art data mining procedure applied herein limited the effect of biases due to data heterogeneity, which commonly affects genome-wide data, and led to a more accurate data interpretation^19,36^. Fourth, functional data interpretation was performed with a ‘competitive’, multivariate method, which ensures to capture consistent and not spurious relationships between phenotypes and genes related to GO-BP/pathways with biological meaning, even for small sample size studies^37–40^. Consistently, we found groups of interconnected pathways that look coherently related to our PAD phenotypes.

This study has also clear limitations. Our PAD patients’ cohort is relatively small, and this is mainly due to the paucity of biopsy material available in current clinical settings. Furthermore, PAD is a group of vascular diseases anatomically characterized by stenosis or occlusion of one or more arteries between the aorta and the upper or lower extremity arteries and, thus, presents a heterogeneous population of patients. For this reason, the differences we revealed in our study between Oc- *versus* St-patients might have been guided by a center-bias selection of the patients. To generalize the findings, our results should be tested on an independent cohort of patients. Finally, we drew our conclusions based on an inferential analysis. Although large-scale and multivariate analyses have been widely shown to be effective in identifying molecular mechanisms related to specific phenotypes, interfering with such mechanisms would likely clarify their putative role in the context of AIOD.

In conclusion, although this is a proof of concept study, our work highlights and confirms the importance of PVAT for the understanding of the underlying pathophysiological mechanisms in abdominal aortic diseases. It would also support the notion that PVAT plays a different pathogenic role in aortic distal atherosclerosis compared to AAA^17^, which are to be considered diseases with different development and evolution mechanisms. Furthermore, it suggests that altered pathways in PVAT, mainly involved in the regulation of lipid metabolism, maintenance and remodeling of the aorta, and immune functions, are functionally associated with different subgroups of PAD patients and may indicate a different natural history of the two conditions. As a clinical exploitation of these findings, peripheral biomarkers could be sought to help distinguish between different PAD patients, e.g. through non-invasive circulating blood testing and/or analysis of easily accessible subcutaneous fat deposits with minimally invasive procedures. This could help to classify (or re-classify) patients showing a different obstructive arteriopathy and characterize their clinical outcomes. Finally, the inferences on specific pathogenic mechanisms presented herein could motivate additional research with potentially clinical significance. For example, genes associated with aorta remodeling, including the TGF-β pathway, can represent possible targets for therapeutic interventions.

## Methods

Anonymized data and materials have been made publicly available at the NCBI’s GEO repository and can be accessed at https://www.ncbi.nlm.nih.gov/geo/query/acc.cgi?acc=GSE136822.

### Study population

Eleven adult patients (>18 years) with PAD, undergoing elective aortoiliac or aortofemoral bypass graft surgery at Centro Cardiologico Monzino between June 2010 and December 2014, were included in the study.

Exclusion criteria included Marfan syndrome and other genetic disorders of the elastic fiber system, active or recent (5 years) cancer, recent major surgery (6 months), aneurysms or disorders of the immune system such as autoimmune diseases or vasculitis. The Ethical Committee of Centro Cardiologico Monzino approved the study and all the participants signed written informed consent.

All research presented herein was conducted according to the principles of the Declaration of Helsinki.

### Sample collection

At the time of surgery, AT samples were collected sequentially (see Piacentini *et al*. for details^17^) as follows: subcutaneous abdominal fat, omental-visceral fat, PVAT surrounding the distal abdominal aorta, and PVAT surrounding the proximal (upper) abdominal aorta free of plaques or thrombus (by angiography and surgeon’s visual inspection at the time of proximal graft anastomosis). Thus, we distinguish the AT surrounding abdominal aorta according to the position of the segment and on the type of lesion, *i.e*.: DAOc- and PxOc-PVAT, and DASt- and PxSt-PVAT. AT samples were promptly snap-frozen in liquid nitrogen and stored at −80 °C until processing.

### Microarray gene expression analysis

The TRIzol Reagent (Thermo Fisher Scientific) was used to extract total RNA from 50–100 mg of frozen samples. To remove genomic contamination, RNAs were treated with TURBO DNase (Thermo Fisher Scientific), following the manufacturer’s instructions. RNA yield/purity and integrity were assessed using the Infinite M200 PRO multimode microplate reader (Tecan) and the 2100 Bioanalyzer (Agilent Technologies), respectively. Of the total 44 AT samples, 1 subcutaneous AT was discarded because of its poor RNA yield and quality. Gene expression assays were performed through the HumanHT‐12 v4 Expression BeadChips (Illumina). RNA isolation and microarray protocols are described in detail in Piacentini *et al*.^17^.

#### Data processing

Array data export and quality control were performed with the Genome Studio Software v2011.1 (Illumina). Raw data were imported into the R software v3.5.0 and normalized with the lumi R/Bioconductor package^41^. Probes with a detection *P* value < 0.01 in at least 30% of the total samples were retained. The *DaMiRseq* R/Bioconductor package was used to identify unwanted sources of variation (aka latent variables) to control the systematic heterogeneity as produced by high-dimensional data^42^. The matrix of expression values was then adjusted for the presence of latent variables by the *DaMiR.SVadjust* function. Microarray probes were annotated through the *lumiHumanIDMapping* and *biomaRt* R/Bioconductor package to retain only those with the most up-to-date annotation^43,44^.

### Statistical analysis

Statistical analysis was carried out in the R environment v3.5.0. Unsupervised clustering analysis of AT samples was performed by PCA using normalized and adjusted expression data.

The *limma* R/Bioconductor package was used to perform differential expression analysis^45^. An additive linear model for a multi-level experiment of paired samples, including “tissue-type” (*ie*, DA-PVAT, Px-PVAT, omental-visceral and subcutaneous AT) and “patient sub-group” (*ie*, those with occlusive or stenotic lesions of abdominal aorta) as factors, was designed as suggested by Smith *et al*.^46^ The implementation of this statistical model, adjusted for the latent variables, allowed computing paired-samples comparisons *within* DA-PVAT and Px-PVAT specimens and *between* the two patient groups.

Transcripts with an absolute log_2_ fold-change | (log_2_FC)| ≥0.38 at a FDR-adjusted *P*-value < 0.05 were reckoned as significantly different. The robustness of the differential expression analysis results was assessed by exploring the histograms of the *P-*value distribution. For truly DE genes, the shape of the histogram should show a uniformly flat distribution across the unit interval (null *P* values) with a peak near zero (*P* values for alternative hypotheses)^47^.

The *sizepower* R/Bioconductor package^48^ was used to assess the power of the differential expression analysis for the two patients’ subgroups. A sample size of n = 5 paired-samples was estimated to provide a statistical power of 90% under certain conditions (see the online-only Data Supplement 1 for parameter details).

### Functional inferences on genome-wide expression profiles

Biological functions associated with the differences observed by differential expression analysis were inferred by taking advantage of prior biological knowledge on genes grouped by GO-BP and by Reactome pathway database (http://www.reactome.org/) using GSEA, v3.0^38^. To reduce redundancy and visually interpret GSEA data, a network of the most significant GO-BP/Reactome pathway gene-sets was drawn through the Enrichment Map software v.3.2.0^49^, implemented as a plug-in in the Cytoscape v.3.7.0 platform^50^.

Details on data processing and analysis parameters are available in the online-only Data Supplement 1.

## Supplementary information

Supplementary information.

Supplementary information2.

Supplementary information3.
