# Supplementary material for: Gene-expression profiles of abdominal perivascular adipose tissue distinguish aortic occlusive from stenotic atherosclerotic lesions and denote different pathogenetic pathways
Source: Sci Rep. 2020 Apr 10;10:6245. doi: 10.1038/s41598-020-63361-5 (PMC7148291; doi:10.1038/s41598-020-63361-5)
Supplement: Supplementary file 1 — Supplementary information. [file 41598_2020_63361_MOESM1_ESM.pdf]

Gene-expression profiles of abdominal perivascular adipose tissue distinguish aortic occlusive from stenotic atherosclerotic lesions and denote different pathogenetic pathways

Luca Piacentini[1]*, Claudio Saccu[3], Elisa Bono[1], Elena Tremoli[4], Rita Spirito[3], Gualtiero Ivanoe Colombo[1†], José Pablo Werba[2†]

[1]Immunology and Functional Genomics Unit, Centro Cardiologico Monzino, IRCCS, 20138 Milan, Italy, [2]Atherosclerosis Prevention Unit, Centro Cardiologico Monzino, IRCCS, 20138 Milan, Italy, [3] Vascular and Endovascular Surgery Unit, Centro Cardiologico Monzino, IRCCS, 20138 Milan [4]Scientific Direction, Centro Cardiologico Monzino, IRCCS, 20138 Milan, Italy.

[†]GIC and JPW provided equal contribution as senior authors.

Supplemental Material

Materials and Methods

Data processing

Global Data normalization. Raw data were analyzed with the *lumi* R/Bioconductor package,[1] which was specifically implemented for processing Illumina microarray. Data variance stabilization was performed by variance stabilizing transformation (VST) and transformed data were normalized by robust spline normalization (RSN) algorithm, which combines the features of quantile and loess normalization. Probes with a detection P-value < 0.01 in at least 30% of the samples were retained.

Data adjustment. Latent variables introduce unwanted heterogeneity in high-throughput data. This unwanted variation can affect both statistical analysis and biological interpretation of the study. Since global data normalization methods may fail to adjust properly the systematic data heterogeneity, the normalized and filtered expression matrix produced by *lumi* method was assessed for the presence of latent (aka surrogate) variables through the *DaMiRseq* R/Bioconductor package.[2] The *DaMiR.SV*() function was set with the “class” variable corresponding to the four different AT and the arguments *method=”fve”*, *th.fve = 0.95* and *second.var* corresponding to the patients’ subgroup (*i.e.* Oc or St). The *second.var* argument is a factor or a numeric vector of an additional variable to take into account during the surrogate variable (SV) identification. This variable together with “class” in the data object was used to design the model matrix (~ class [AT] + second.var [Oc-St]). Six putative surrogate variables (SV) were identified. The first three SV associated with technical variability and other putative unknown confounding (latent) factors and were subsequently included in the statistical model design to adjust the differential expression analysis. The forth to sixth SV were excluded from the statistical model as they presented a low but significant association with the patients’ class or subgroup factors, which are patient’s variables that we took into consideration for the following differential expression analysis.[3]

Probe annotation. Microarray probes were firstly annotated through the *lumiHumanIDMapping* R/Bioconductor package.[4] Probes were further filtered by the *biomaRt* R/Bioconductor package[5] to retain only those probes corresponding to genes with a stable annotation and avoid the inclusion of non-technically supported or deprecated sequences. The *biomaRt* query functions were set to select the “ensemble” database and the “hsapiens_gene_ensembl” (version GRCh38.p10) dataset, and filter Illumina probes on the bases of the NCBI Reference Sequence Database (RefSeq) of coding (“refseq_mrna”) and non-coding (“refseq_ncrna”) annotated RNAs.

Statistical power calculation parameters. Power calculation was performed by the *sampleSize.matched()* function of the *sizepower* R/Bioconductor package.[6] Arguments of the functions were set as follows: mean number of false positives (ER0) =14; anticipated number of genes in the experiment that are not differentially expressed (G0) =14000; absolute mean difference in log-expression (absMu1) = 0.38; anticipated standard deviation (sigmad) = 0.20 (with sigma derived from data of this study); and power=0.9. The required sample size for a statistical power of 90% was of at least 5 paired-samples. Since the vast majority of the observed significant DE genes in all the comparisons had a log2_FC >0.45, we calculated that a slight increase of absMu1 to 0.45 raised the power for the Oc (n=6) and St (n=5) paired-sample size to 98% and 95%, respectively.

Functional inferences on genome-wide expression profiles

GSEA parameters. Gene sets used for GSEA were retrieved from the repository of the Bader Lab gene-set collections (http://download.baderlab.org/EM_Genesets/). The GO-BP and Reactome gene-sets were merged into a unique Gene Matrix Transposed file format (*.gmt) to perform a single GSEA analysis. The GSEA pre-ranked tool option was adopted. A combined gene rank-score, calculated as the product of the log2 fold-change x -log10(P-value) obtained by the differential expression analysis, was used as the gene-ranking metric. The combined gene rank-score was applied to weigh the relevance of the gene by taking into consideration both the amplitude (*i.e.* fold-change) and the statistical consistency of the gene expression differences. Other GSEA parameters included 10000 permutations and gene-sets size limit ranging from 8 to 150 genes. The threshold for statistical significant gene-sets was set at an FDR<0.05.

Supplementary Figure I

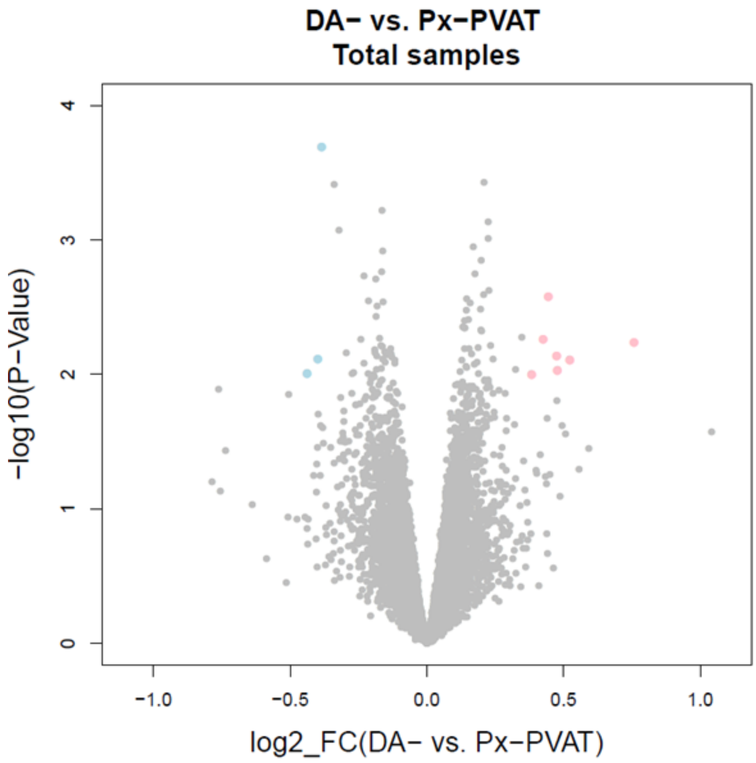

Suppl. Figure I. Differential expression analysis on total paired samples. Scatterplot of the log2 FC *vs.* the significance (x-and y-axis, respectively) for the comparison of the DA- versus Px-PVAT in total samples. Pink and light blue dots represent DE transcripts at nominal P-Value<0.01, with an absolute log2 FC ≥0.38 but that did not stand adjustment for multiple testing (adj.P Value<0.05).

Supplementary Figure II

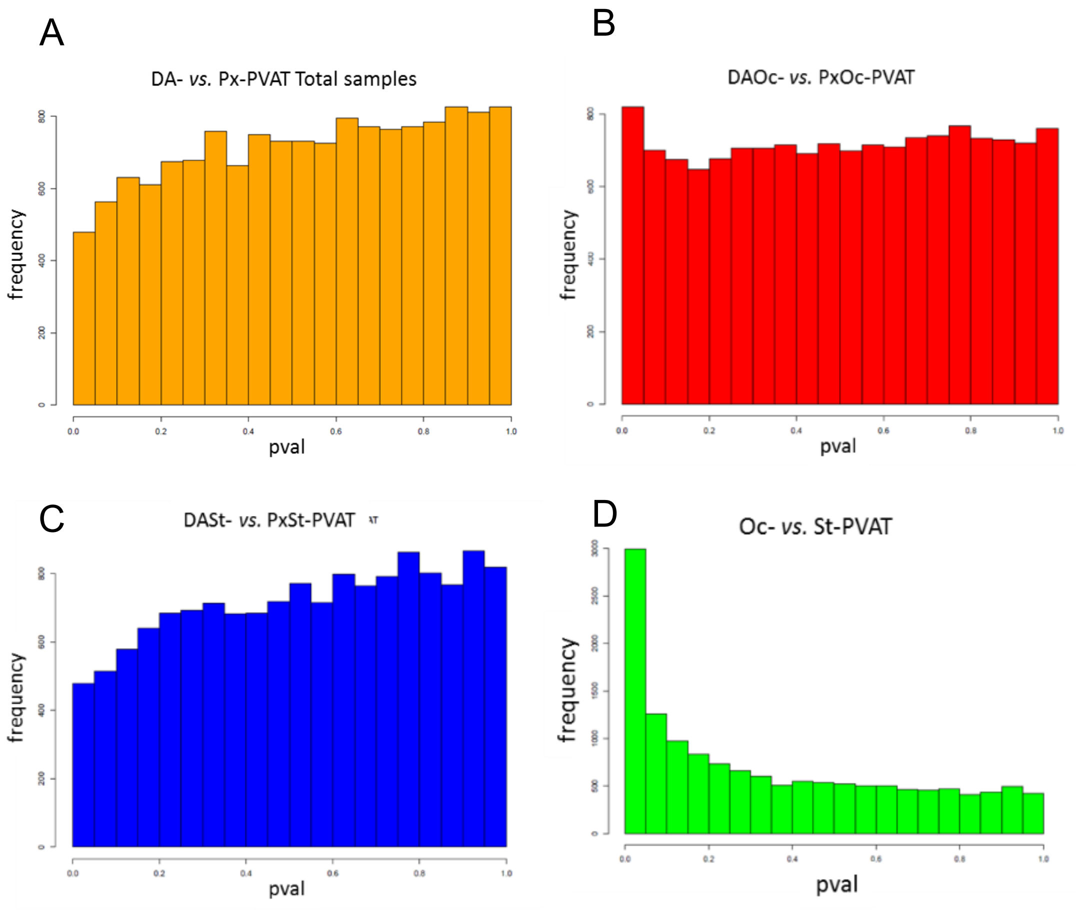

Suppl. Figure II. Histograms of P values distribution. The histogram of the P values distribution for non-DE transcripts is ideally uniformly distributed across the unit interval (null hypothesis) with a spike near zero for truly DE transcripts (alternative hypothesis). Histogram for *within* comparison of DA- *vs.* Px-PVAT on total samples (**A**), DAOc- *vs.* PxOc-PVAT (**B**), DASt- *vs.* PxSt-PVAT (**C**) and *between* PVAT of Oc- *vs.* St-patients (**D**) are shown.

Supplementary Figure III

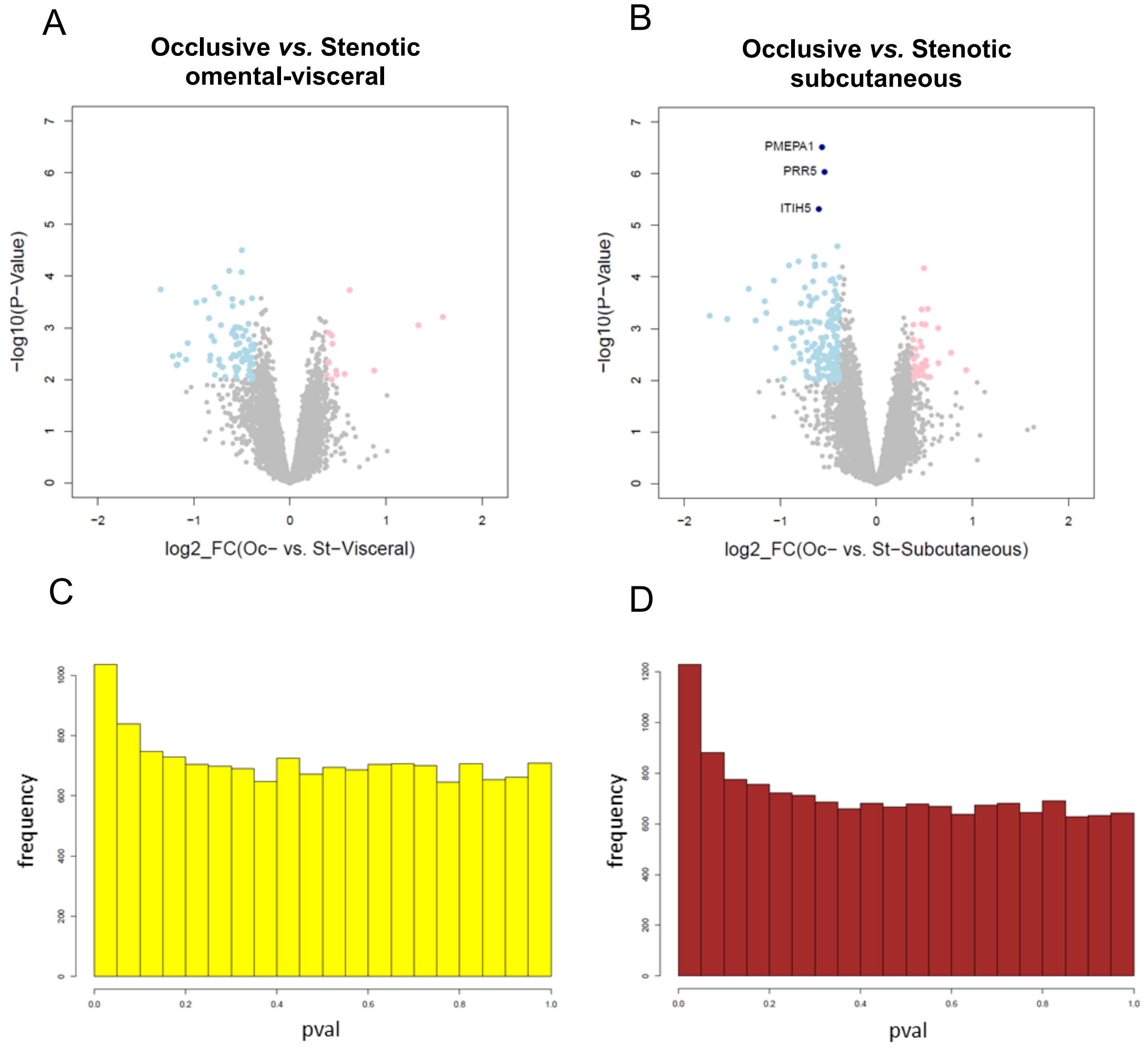

Suppl. Figure III. Differential expression analysis for omental-visceral and subcutaneous AT. Scatterplot of the log2 FC vs. the significance (x-and y-axis, respectively) for the Oc- vs. St-patients in omental-visceral (**A**) and subcutaneous (**B**) AT. Pink and light blue dots represent significant DE transcripts at nominal P-Value<0.01, with an absolute log2 FC ≥0.38. Dark blue dots represents DE transcripts that stood adjustment for multiple testing (adj.P-Value<0.05). On the lower panel, the histograms of the P Values distribution for omental-visceral (**C**) and subcutaneous (**D**) AT comparisons *between* Oc- and St-patients are shown. P values distribution displayed the expected shape for truly DE transcripts for both the two comparisons.

Supplementary Figure IV

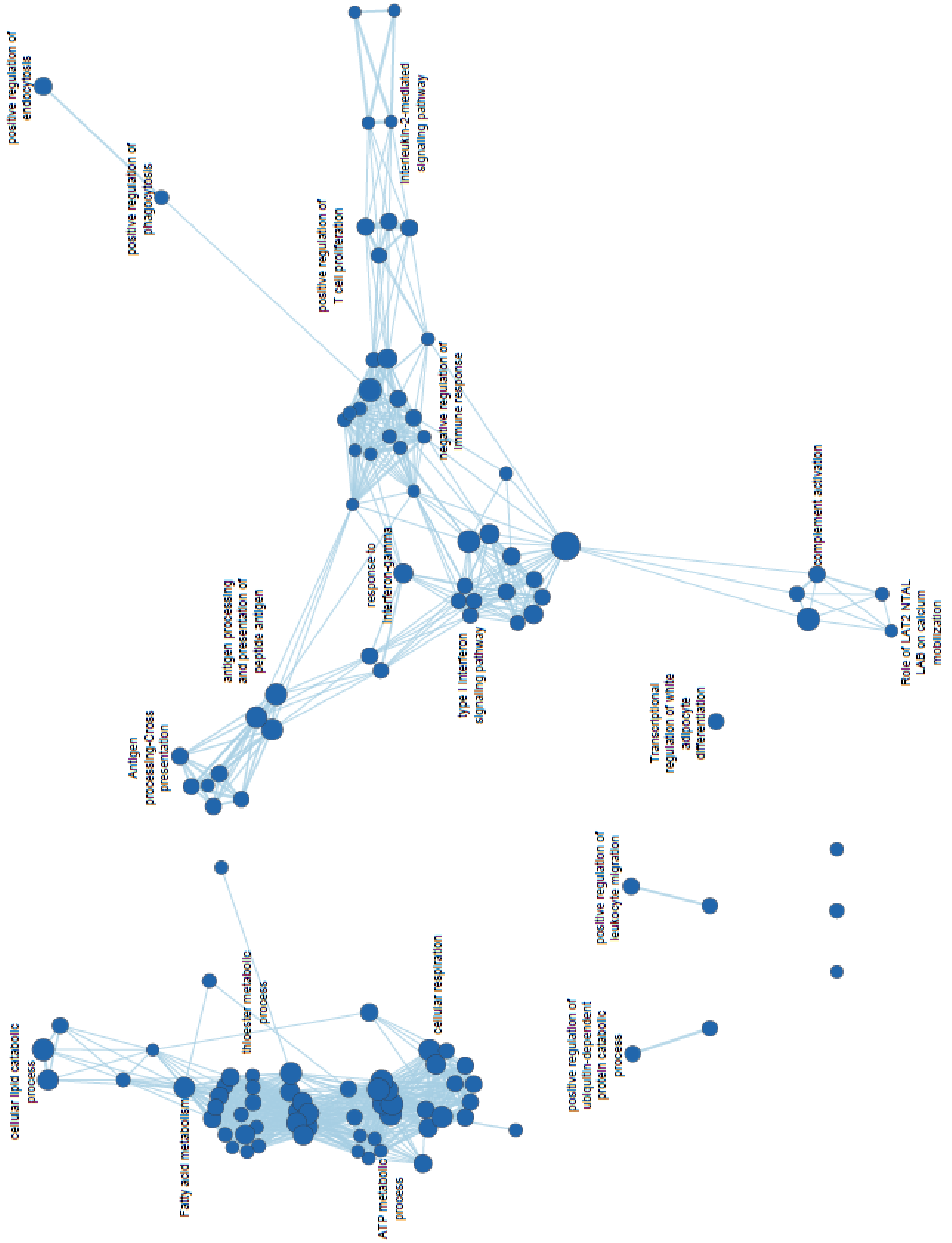

Suppl. Figure IV. Enrichment map for Oc- vs.St-omental-visceral comparison. The enrichment network shows the pathway/GO-BP gene-sets (nodes) that are significantly associated (FDR<0.05) either with Oc- *or* St- omental-visceral. Legend scheme as in Figure 4 of the manuscript. Supplemental material 3b reported the complete results of the enrichment analysis by GSEA used to draw the network. Enrichment network was drawn using the Enrichment Map software v.3.2.0[7], implemented as a plug-in in the Cytoscape v.3.7.0 platform[8].

Supplementary Figure V

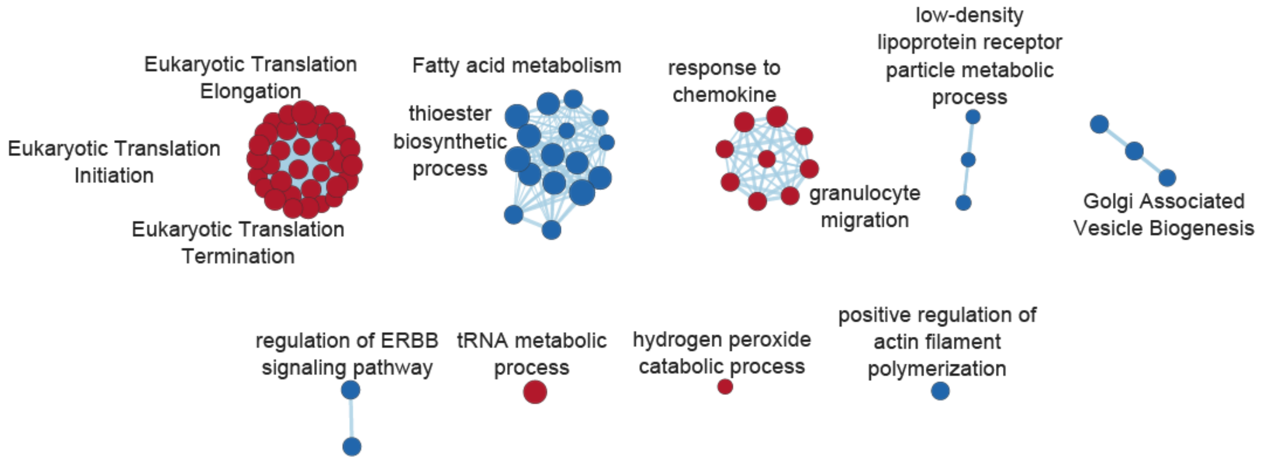

Suppl. Figure V. Enrichment map for Oc- vs.St-subcutaneous. The enrichment network shows the pathway/GO-BP gene-sets (nodes) that are significantly associated (FDR<0.05) either with Oc- *or* St- subcutaneous AT. Legend scheme as in Figure 4 of the manuscript. Supplemental material 3c reported the complete results of the enrichment analysis by GSEA used to draw the network. Enrichment network was drawn using the Enrichment Map software v.3.2.0[7], implemented as a plug-in in the Cytoscape v.3.7.0 platform[8].

References

1. Du, P., Kibbe, W. A. & Lin, S. M. lumi: A pipeline for processing Illumina microarray. Bioinformatics **24**, 1547–1548 (2008).

2. Chiesa, M., Colombo, G. I. & Piacentini, L. DaMiRseq -An R/Bioconductor package for data mining of RNA-Seq data: Normalization, feature selection and classification. Bioinformatics **34**, 1416–1418 (2018).

3. Ritchie, M. E. et al. limma powers differential expression analyses for RNA-sequencing and microarray studies. Nucleic Acids Res. **43**, e47 (2015).

4. Du, P., Feng, G., Kibbe, W. & Lin, S. lumiHumanIDMapping: Illumina Identifier mapping for Human. (2016). doi:10.18129/B9.bioc.lumiHumanIDMapping http://bioconductor.org/packages/release/data/annotation/manuals/lumiHumanIDMapping/man/lumiHumanIDMapping.pdf. Accessed July 27, 2018.Google Scholar

5. Durinck, S. et al. BioMart and Bioconductor: A powerful link between biological databases and microarray data analysis. Bioinformatics **21**, 3439–3440 (2005).

6. Qiu, W., Lee, M. T. & Whitmore, G. A. Sample Size and Power Calculation in Microarray Studies Using the sizepower package . R package version 1.32.0 (2006). doi:10.1080/10543406.2010.500066. https://bioconductor.org/packages/release/bioc/vignettes/sizepower/inst/doc/sizepower.pdf. Accessed November 15, 2018. Google Scholar.

7. Merico, D., Isserlin, R., Stueker, O., Emili, A. & Bader, G. D. Enrichment map: A network-based method for gene-set enrichment visualization and interpretation. PLoS One **5**, e13984 (2010).

8. Shannon, P. et al. Cytoscape: A software Environment for integrated models of biomolecular interaction networks. Genome Res. **13**, 2498–2504 (2003).
